# A novel RLBP1 gene geographical area-related mutation present in a young patient with retinitis punctata albescens

**DOI:** 10.1186/s40246-017-0114-6

**Published:** 2017-08-01

**Authors:** Concetta Scimone, Luigi Donato, Teresa Esposito, Carmela Rinaldi, Rosalia D’Angelo, Antonina Sidoti

**Affiliations:** 1 Department of Biomedical and Dental Sciences and Morphofunctional Imaging, Division of Molecular Genetics and Preventive Medicine, University of Messina, via C. Valeria 1, I-98125 Messina, Italy; 2 Department of Cutting-Edge Medicine and Therapies, Biomolecular Strategies and Neuroscience, Section of Neuroscience-applied Molecular Genetics and Predictive Medicine, I. E. ME. S. T, via Michele Miraglia 20, I-90139 Palermo, Italy; 3 Department of Experimental Medicine, Division of Human Physiology and Integrate Biological Functions “F. Bottazzi”, University of Campania Luigi Vanvitelli, ex II University of Naples, via Santa Maria di Costantinipoli 16, I-80138 Naples, Italy

**Keywords:** Retinitis punctata albescens, RLBP1, Frameshift mutation, Population study, Geographical-area related mutation, RP mutation spectrum

## Abstract

**Background:**

Autosomal recessive forms of retinitis punctata albescens (RPA) have been described. RPA is characterized by progressive retinal degeneration due to alteration in visual cycle and consequent deposit of photopigments in retinal pigment epithelium. Five loci have been linked to RPA onset. Among these, the retinaldehyde-binding protein 1 gene, RLBP1, is the most frequently involved and several founder mutations were reported. We report results of a genetic molecular investigation performed on a large Sicilian family in which appears a young woman with RPA.

**Results:**

The proband is in homozygous condition for a novel *RLBP1* single-pair deletion, and her healthy parents, both heterozygous, are not consanguineous. Thenovelc.398delC (p.P133Qfs*258) involves the exon 6 and leads to a premature stop codon, resulting in a truncated protein entirely missing of CRAL-TRIO lipid-binding domain.

Pedigree analysis showed other non-consanguineous relatives heterozygous for the same mutation in the family. Extension of mutation research in the native town of the proband revealed its presence also in healthy subjects, in a heterozygous condition.

**Conclusions:**

A novel *RLBP1* truncating mutation was detected in a young girl affected by RPA. Although her parents are not consanguineous, the mutation was observed in a homozygous condition. Being them native of the same small Sicilian town of Fiumedinisi, the hypothesis of a geographical area-related mutation was assessed and confirmed.

## Background

Retinitis pigmentosa (RP) includes more than 70 different forms of inherited eye disorders characterized by progressive vision loss due to photoreceptors degeneration. This heterogeneity is caused by the high number of genes involved in disease’s development. Therefore, each RP form differs from another for causative gene, inheritance pattern, symptomatology, age onset, and clinical features. About genetics, more than 50% of cases are affected by autosomal recessive forms due to mutations in almost 50 loci [1]. Among these, the *retinaldehyde-binding protein 1* (*RLBP1*) mutations are cause of retinitis punctata albescens (RPA, OMIM#136880) [2]. RPA is a progressive retinal degeneration belonging to the group of rod-cone dystrophies and has an early onset characterized by night blindness. At diagnosis, fundus appears with punctate white–yellow deposits. Progressive macular atrophy is the main cause of visual acuity loss, and narrowing of the visual field may occur in late teenage [3]. RPA has an incidence of 1/800000 people worldwide, and mutations at RLBP1 gene were reported only in about 1% of patients affected by autosomal recessive forms [2]. However, RPA cases associated with *Rhodopsin* (*RHO*), *Retinol Dehydrogenase 5* (*RDH5*), *Peripherin 2* (*PRPH2*), and *Lecithin Retinol Acyltransferase* (*LRAT*) genes were also reported [4–6]. About RLBP1 gene, different mutations are linked to a wider spectrum of phenotype as Bothnia retinal dystrophy (BD), Newfoundland rod-cone dystrophy (NFRCD), and fundus albipunctatus (FA). These forms differ for age of onset, progression, and for severity [7]. A founder effect was proven for mutation p.R234W, causing Bothnia phenotype [8], as well as for splice-junctions mutations detected in NFRCD cases [9]. So, different clinical manifestations are tightly linked to the effects of mutations on protein’s structure. *RLBP1* is expressed in retinal pigment epithelium (RPE) and encodes for the cellular retinaldehyde-binding protein 1, CRALBP, involved in visual cycle. Particularly, CRALBP binds 11-cis-retinol which needs to be oxidized in 11-cis-retinal; 11-cis-retinal is carried into photoreceptors where it combines with opsin, resulting in visual pigments formations [10]. This process is common in both cones and rods.

We describe a novel *RLBP1* frameshift mutation, detected in a homozygous condition in a young 31-years-old woman, affected by RPA, certainly with no consanguineous parents up to the 4th generation ancestor, and native in Fiumedinisi, a small Sicilian town in province of Messina. Fiumedinisi is entered in a valley of Nebrodi mountains, at a height of about 200 above sea level. Homozygous condition for a novel mutation is a rare event in patients whose parents are not consanguineous; however, in this case, parents are both native of Fiumedinisi. This leads us to hypothesize that detected mutation is a geographical area-related variant.

## Methods

### Case analysis

#### Case description

**Fig. 1:**
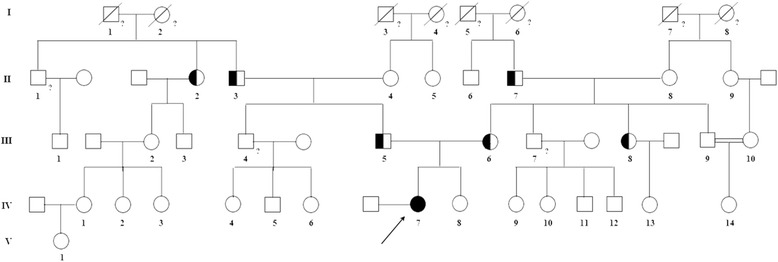
Familial pedigree. The *arrow* indicates the proband (*IV:7*). *Empty squares* and *circles* symbolize wild-type men and women, respectively. *Half fill* indicates the heterozygous condition for the c.398delC mutation. *Question mark* indicates the unknown genotype due to unavailability of the sample. *Slash* indicates died individuals

**Fig. 2:**
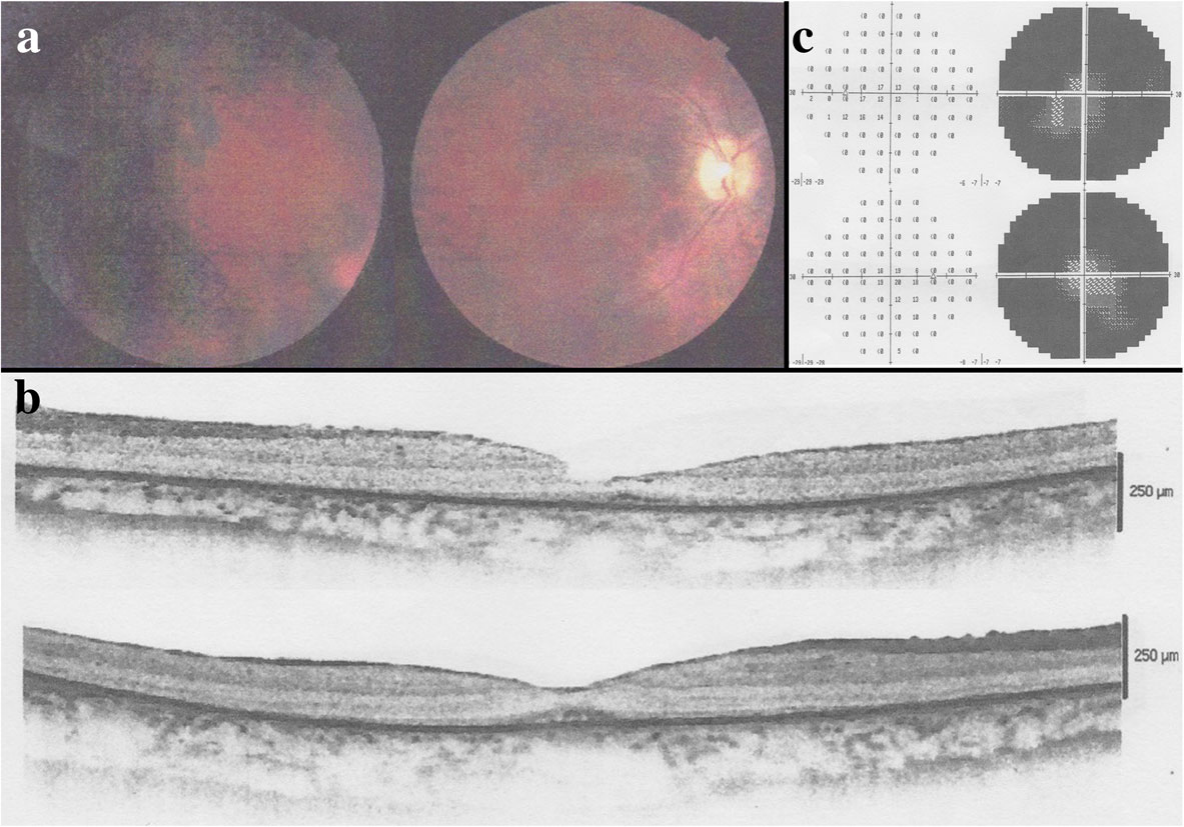
Clinical investigations. Fundus photograph of proband’s both eyes (**a**) shows papillary pallor, fundus albipunctatus, narrowed retinal arteries and temporal displacement of the papilla opaque vessels. A horizontal SD–OCT scan (**b**) shows numerous well-demarcated homogenous dome-shaped lesions originating from the RPE layer and diffused to the IS/OS junction of the photoreceptors, external limiting membrane, and into the outer nuclear layer. Visual field of both eyes (**c**) shows adeep impairment of visual field from tapeto-retinal degeneration; GHT’s 30° Threshold Test denotes annular scotom in the explored area with central vision islands

Here we present a case of a 31-years-old woman (Fig. 1, IV:7), born in Fiumedinisi, a small Sicilian (Italy) town. Her parents, also native of Fiumedinisi, are not consanguineous. About the proband, at the age of five, myopia was diagnosed; two years later, she began suffering from night vision disturbance. Fundus revealed the typical “salt and pepper” aspect (Fig. 2a), without macula involvement; additionally, optical coherent tomography (OCT) examination showed the presence of numerous well-demarcated homogenous dome-shaped lesions, originating from the RPE layer (Fig. 2b). One year later, fundus analysis confirmed the progressive kind of phenotype, so RPA was diagnosed. Thereafter, she has report continuous worsening with also reduction of the visual field due to “retinal-tapetum” degeneration (Fig. 2c). No evidences of eye diseases were reported neither for her parents nor for younger sister.

#### Molecular analysis

DNA was extracted from peripheral blood. Exons and exon-intron bourdaries of *RLBP1*, *PRPH2*, *RHO*, *LRAT*, and *RDH5* genes were amplified by polymerase chain reaction (PCR). Primers sequences and PCR conditions are available upon request. Direct sequencing was performed using BigDye Terminator v3.1 chemistry and sequencing was ran on a 3500 Genetic Analyzer (Applied Biosystems). Molecular screening of all genes was performed in the proband; then, detected variants were also searched in the other family members.

#### In-silico prediction

Effects of the novel mutation on CRALBP tertiary structure were predicted by RaptorX (http://raptorx.uchicago.edu/) prediction tool.

### Population study

#### Samples collection

To test the frequency of novel c.398delC variant, two groups made up by 300 and 600 subjects, respectively, heterogeneous for age and gender, were collected. The first one was recruited in Fiumedinisi and sampling criteria include Fiumedinisi native ancestors by at least three generations, absence of consanguinity, and absence of inherited disabling ocular diseases. The second group was collected in the Sicilian area: Ionic and Tyrrhenian municipality in province of Messina, like Santa Teresa Riva, Roccalumera, Pace del Mela, were excluded due to their geographic localization, since destinations of the inhabitants of Fiumedinisi who have left their native town. For each population, the c.398delC allele frequency was calculated as [1 × (h + 2H)]/2 *N*, where *h* represents the heterozygous genotype, *H* thehomozygous genotype, and *N* the sample size for each population. Deviation from Hardy-Weinberg equilibrium (HWE) was determined using the *χ* ^**2**^test with 2 × 3 contingency tables and 1 degree of freedom. Analysis was performed by the IBM SPSS statistical analysis software.

#### Molecular analysis

Buccal swabs were used for collecting saliva samples. DNA was purified by QIAamp® DNA Mini kit (Qiagen). Exon 6 of RLBP1 gene was amplified by PCR for all collected samples, then sequenced on 3500 Genetic Analyzer (Applied Biosystems), using BigDyeTermitor v3.1 chemistry.

All patients and controls involved in this study were fully informed and written consent was obtained. For underage subjects, consent was obtained from the parents. The study protocol followed the guidelines of our local ethics committee, and the investigation was conducted with the ethical requirements defined in the Helsinki Declaration.

## Results

### Case analysis

**Fig. 3:**
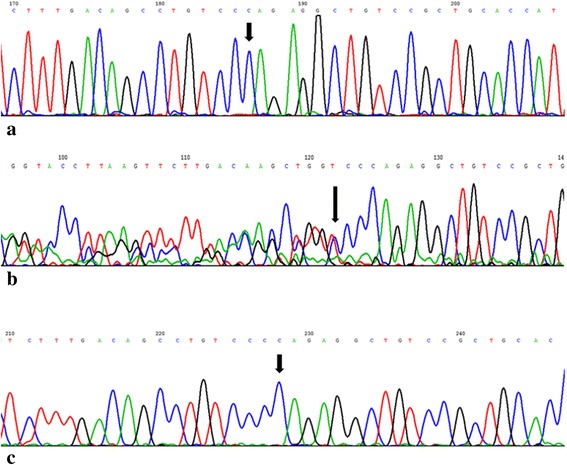
Partial electropherograms of *RLBP1* exon 6. The *arrows* indicate affected nucleotide. **a** Electropherogram showing the homozygous condition for c.398delC mutation. **b** Electropherogram showing the heterozygous condition, sequence in reverse. **c** Wild-type sequence

**Fig. 4:**
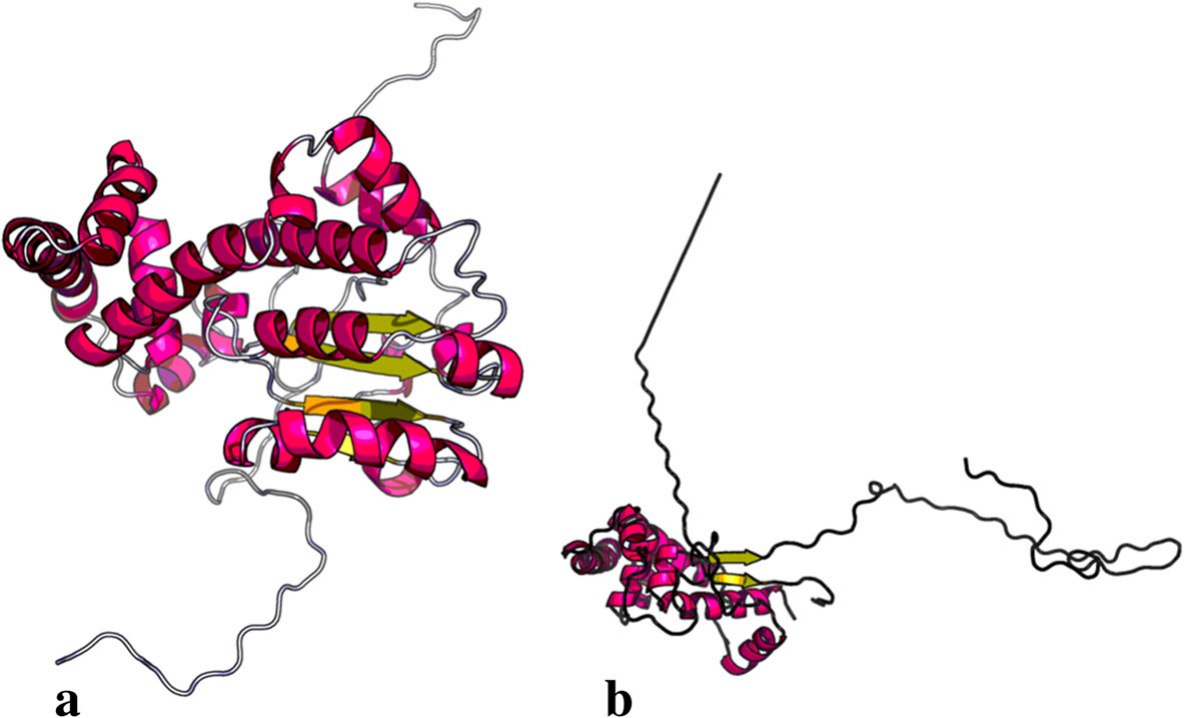
CRALBP Tertiary structure alteration prediction. Structural models were generated by RaptorX tool. **a** Tertiary structure of wild-type protein. **b** Tertiary structure of p.P133Qfs*258 affected protein

Direct sequencing of all known RPA causative genes *LRAT*, *RDH5*, and *RHO* showed no significant variants. Two SNPs rs390659 (c.910C > G, p.Q304E) and rs434102 (c.1013A > G, p.D338G) were detected in *PRPH2*; however, in ClinVar database, these are reported as benign (http://www.ncbi.nlm.nih.gov/clinvar/variation/138904/, http://www.ncbi.nlm.nih.gov/clinvar/variation/138906/). A novel single base-pair frameshift deletion, the c.398delC (p.P133Qfs*258), was detected in exon 6 of RLBP1 gene in a homozygous condition (Fig. 3a). It is not present in mutations and SNPs databases as HGMD, dbSNPs, ClinVar, ExAC, and Ensembl. The variant leads to substitution of proline 133 in glutamine and results in a frameshift with consequent premature termination at the 258th codon, in exon 8, causing the loss of the last 60 amino acids at the C-terminus of the protein. Figure 4 shows the results of in-silico prediction of tertiary structure alteration in mutated protein (Fig. 4b) compared to wild-type one (Fig. 4a). As evident, the c.398delC results in total disruption of functional domain as well as in an altered folding.

About other family’s members, the novel mutation was observed in both parents (Fig. 1, III:5, III:6), in heterozygous condition (Fig. 3b) and was absent in the sister (Fig. 1, IV:8; Fig. 3c). Moreover, mutated allele was found both in paternal and in maternal relatives (Fig. 1, II:2, II:3, II:7, III:8).

#### Population study

As previously described, *RLBP1* mutations are very uncommon and founder mutations were reported. Frequency of c.398delC allele was assessed in both Fiumedinisi and Sicilian population. In the first group, the mutated allele was detected in 0.01%, while none of screened samples of general Sicilian population showed the variant. Table 1 shows the distribution of allele frequencies in Fiumedinisi population; *χ* ^2^ test results in a *p* value = 0.9306 showing the condition of HWE (HWE not consistent for *p* < 0.05).

**Table 1:** Wild-type (+) and c.398delC (−) allele frequencies in Fiumedinisi population

	Samples	Allele +	Allele −	Observed frequency	%	HW-expected frequency	%	Chi-square	*p* value
**+/+**	297	594	0	297	99.00	297.01	99.00	0.000	
**+/−**	3	3	3	3	1.00	2.99	1.00	0.000	
**−/−**	0	0	0	1	0.00	0.01	0.00	0.008	
Total	300	597	3	300	100.00	300.00	100.00	0.008	*0.9306*
	600	*1.00*	*0.01*	600				(*p* value) Chisq w 1 df	

A total of 300 samples were genotyped. Only 0.01% of screened population carries the mutated allele. Chi-square test exhibits a *p* value = 0.9306 suggesting the presence of the equilibrium’s condition between wild-type and mutated allele in Fiumedinisi population

## Discussion

This study reports the results obtained by a molecular analysis performed on a large Sicilian family in which a case of RPA was diagnosed. Molecular screening of all known RPA causative genes showed a novel single base-pair frameshift deletion, c.398delC, in exon 6 of *RLBP1*. It leads to a truncated 258 amino acid protein. Premature stop codon falls within exon 8. CRALBP protein contains one CRAL-TRIO lipid-binding domain, of 162 aminoacids (136-297). As predicted by in-silico analysis, the c.398delC affects proline 133 and causes total disruption of CRAL-TRIO domain, resulting in a loss of ability to bind its ligands. CRAL-TRIO motifs usually bind small hydrophobic molecules. In CRALBP, aminoacids 136-297 form CRAL-TRIO domain and, 12 of these, form the retinoid binding pocket [11]. Therefore, c.398delC has deleterious effects on protein’s functionality.

**Fig. 5:**
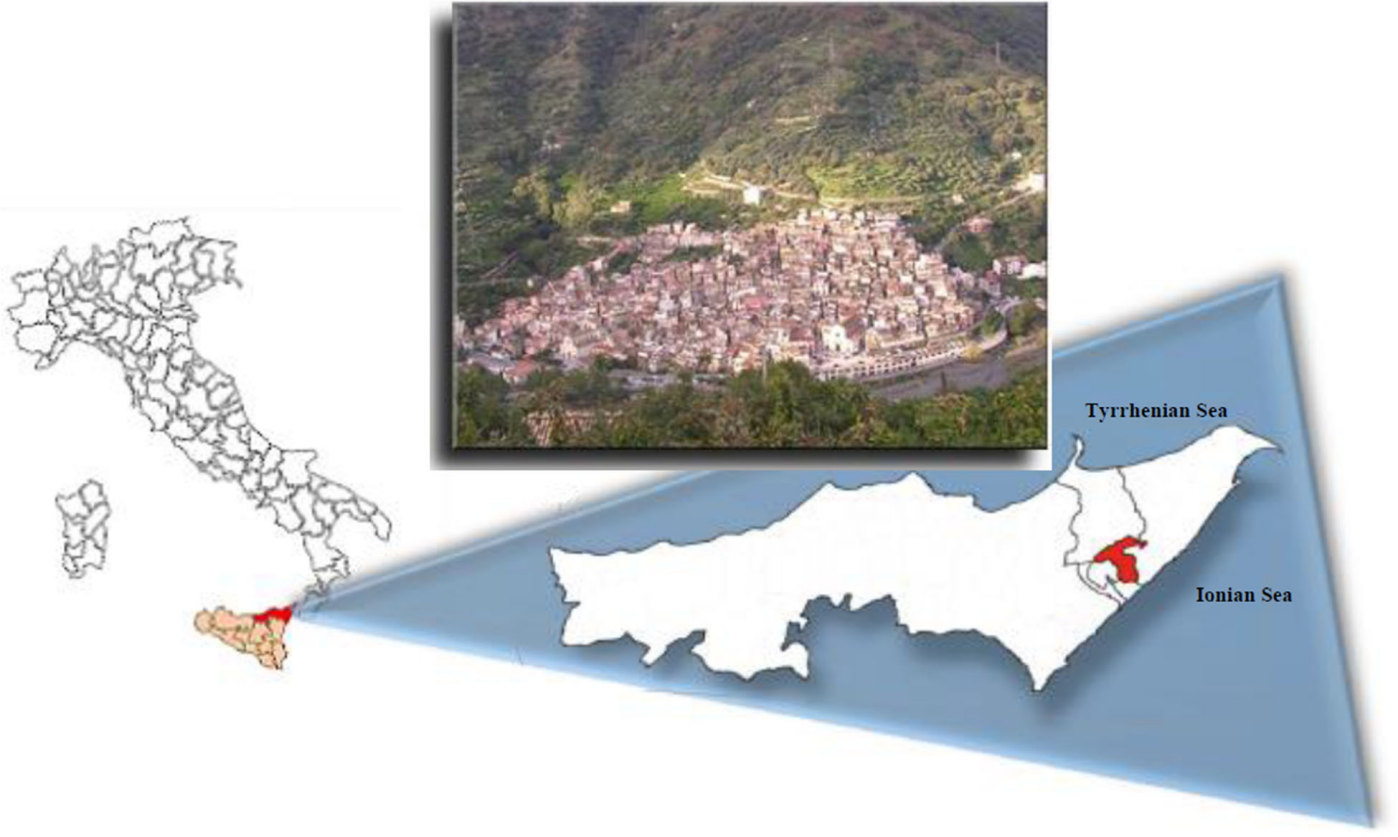
Focus of geographical area. On the leftmost are showed Italy and Sicily (*pink*); province of Messina is *red* highlighted and enlarged on the right of the picture. Red municipality is Fiumedinisi. Delimited areas are “Valle del Mela” and Nizza, Santa Teresa Riva and Roccalumera, respectively, on the Tyrrhenian and Ionian sides. The *upper picture* is an aerial view of Fiumedinisi

Presence of variant c.398delC was assessed also in the other family’s members; despite, they show no pathological phenotypes. Of these, the healthy father and other relatives showed this variant, in a heterozygous condition. This confirms autosomal recessive model of inheritance of RPA. The proband carries the novel c.398delC mutation in a homozygous condition. We proceeded to investigate frequency of variant in Fiumedinisi’s population. Fiumedinisi, a small town in province of Messina, is located on the Ionian side, at the height of 190 above sea level (Fig. 5). It has an area of 36 km^2^ and extends along Nisi’s valley, at Mt. Belvedere’s slopes, behind Valle del Mela. The city was founded in VII century B.C., by a group of Calcidesi Greek colonists, attracted by mineral deposits, who inhabited the high area of Mt. Belvedere. Later, Roman, Arab, Norman, Angevin, and Spanish dominions went on. During these centuries, the town was moved to the foot of mountain (where it remains today) and renamed as FlumenDionisyi. Still today, it results in not being easy to get to. Two big disasters hit the city: an epidemic plague in 1743 and a big flood in 1855. A period of decline, then, started due to high immigration’s phenomenon. To date, Fiumedinisi’s population counts about 1400 inhabitants, most of which are over the age of 50 years. For our aim, we screened about 25% of population and c.398delC was found in 0.01% and it is in HWE. No other RPA cases were detected in the town. Although there is a high rate of consanguinity in Fiumedinisi’s population, its low frequency may be attributed to several dominations, repeated drastic reductions in population, the probable selective disadvantage, and a recent founder effect. This last hypothesis can be demonstrated by several statistic approaches. Among these, the method proposed by Slatkin and Rannala [12, 13] based on allele frequency and coalescent process [14] is what we think to apply to estimate the age of c.398delC mutation, as next development of this paper.

## Conclusions

We report a case with RPA caused by the novel mutation, the c.398delC, in RLBP1 gene in homozygous condition necessary for developing the disease. The new mutation enriches the already wide spectrum of RP causative mutations. Hypothesis of a geographic area-related mutation was induced by the detection of this allele in several non-consanguineous inhabitants and by its absence in Sicilian population. However, due to the succession of more dominations, it is very difficult to establish by which ancestral population it was introduced.

## Abbreviations

CRALBP: Cellular retinaldehyde-binding protein
FA: Fundus albipunctatus
HWE: Hardy-Weinberg Equilibrium
NFRCD: Newfoundland rod-cone dystrophy
OCT: Optical coherent tomography
RLBP1: Retinaldehyde binding protein 1
RP: Retinitis pigmentosa
RPA: Retinitis punctata albescens
RPE: Retinal pigment epithelium
